# An application based on bioinformatics and machine learning for risk prediction of sepsis at first clinical presentation using transcriptomic data

**DOI:** 10.3389/fgene.2022.979529

**Published:** 2022-09-02

**Authors:** Songchang Shi, Xiaobin Pan, Lihui Zhang, Xincai Wang, Yingfeng Zhuang, Xingsheng Lin, Songjing Shi, Jianzhang Zheng, Wei Lin

**Affiliations:** ^1^ Department of Critical Care Medicine, Shengli Clinical Medical College of Fujian Medical University, Fujian Provincial Hospital South Branch, Fujian Provincial Jinshan Hospital, Fujian Provincial Hospital, Fuzhou, China; ^2^ Department of Critical Care Medicine, Shengli Clinical Medical College of Fujian Medical University, Fujian Provincial Hospital, Fuzhou, China; ^3^ Department of Orthopedics, Shengli Clinical Medical College of Fujian Medical University, Fujian Provincial Hospital, Fuzhou, China; ^4^ Department of Endocrinology, Shengli Clinical Medical College of Fujian Medical University, Fujian Provincial Hospital, Fuzhou, China

**Keywords:** application, prediction, transcriptome, sepsis, machine learning

## Abstract

**Background:** Linking genotypic changes to phenotypic traits based on machine learning methods has various challenges. In this study, we developed a workflow based on bioinformatics and machine learning methods using transcriptomic data for sepsis obtained at the first clinical presentation for predicting the risk of sepsis. By combining bioinformatics with machine learning methods, we have attempted to overcome current challenges in predicting disease risk using transcriptomic data.

**Methods:** High-throughput sequencing transcriptomic data processing and gene annotation were performed using R software. Machine learning models were constructed, and model performance was evaluated by machine learning methods in Python. The models were visualized and interpreted using the Shapley Additive explanation (SHAP) method.

**Results:** Based on the preset parameters and using recursive feature elimination implemented via machine learning, the top 10 optimal genes were screened for the establishment of the machine learning models. In a comparison of model performance, CatBoost was selected as the optimal model. We explored the significance of each gene in the model and the interaction between each gene through SHAP analysis.

**Conclusion:** The combination of CatBoost and SHAP may serve as the best-performing machine learning model for predicting transcriptomic and sepsis risks. The workflow outlined may provide a new approach and direction in exploring the mechanisms associated with genes and sepsis risk.

## 1 Introduction

Advances in gene sequencing have generated a large number of omics datasets, including that for the genome, transcriptome, proteome, metabolome, and others (Mirza et al., 2019). Genomics analysis has shown that differential expression of RNA or DNA variants may contribute to the risk of disease (Wray et al., 2021). The association of genes with disease phenotypes has been studied extensively and provides insights into the associated etiological mechanisms (Berndt et al., 2013). However, there are challenges in linking the changes related to the genotype and the resultant phenotypic traits.

Most of the current methods for screening target genes utilize differential gene expression analysis, dimensionality reduction, or correlation. Evidence indicates that a crucial proportion of phenotypic variation cannot be explained by analyzing significance (Dudbridge, 2013), and this approach has resulted in restricting analyses and a limited ability to detect low-risk variants at stringent significance levels.

Machine learning has garnered considerable attention owing to its excellent ability to deal with predictive analysis. Models of risk scores, built using machine learning methods via quantitative analysis of gene expression, can differentiate disease states from controls across transcriptomic datasets (Cao et al., 2018). Compared to classical methods, recent studies have shown the utility of machine learning in the analysis of high-dimensional datasets (Hoadley et al., 2014; Cao et al., 2018; Cheng et al., 2021).

Although machine learning has shown promise in assisting humans in the analysis of a wide variety of datasets related to genomics and genetics, the data obtained must be analyzed, interpreted, and acted upon (Libbrecht and Noble, 2015; Obermeyer and Emanuel, 2016). A few challenges are associated with approaches based on machine learning, such as preprocessing and selection of the relevant gene, construction and evaluation of the model, and the limited interpretability of the model (Ching et al., 2018; Katsaouni et al., 2021). In this study, we developed a bioinformatics and machine learning-based workflow to predict the risk of sepsis using transcriptomic data obtained after the first clinical presentation. By combining bioinformatics with machine learning methods, we have attempted to solve a few of the current challenges in predicting disease risk using transcriptomic data.

## 2 Methods

### 2.1 Study design

The data was obtained from the Gene Expression Omnibus (GEO) database at the National Center for Biotechnology Information. The expression dataset GSE185263 was obtained from the GPL16791 platform (Illumina HiSeq 2,500) contributed by Baghela et al. (Baghela et al., 2022). Diverse cohorts from emergency rooms (ER) of five hospitals and one intensive care unit (ICU) (>18 years of age) with suspected cases of sepsis were included in the study. We performed whole blood RNA-seq and machine learning analysis to develop a transcriptomic gene expression signature for predicting sepsis. Identification of markers predicting the state of sepsis within the first hours of ER may prevent progression to severe sepsis promptly.

### 2.2 Definition

Patients were recruited within 2 h of emergency admission if sepsis was suspected. The enrolled patients required the prevalence of at least two Systemic Inflammatory Response Syndrome/sepsis-1 criteria (Bone et al., 1992) and suspected infection. Based on the sepsis-3 criteria (Shankar-Hari et al., 2016), infection was not confirmed, but the individuals were considered to be at risk for sepsis. Patients with suspected pulmonary sepsis in the ICU were enrolled within the first day or prospectively in the COLOBILI study. Patients were excluded from the study if death occurred within 12 h, a blood sample was not obtained, or consent was denied. No attempt at correction for treatments was performed.

### 2.3 Data collection

A total of 348 samples related to sepsis were recruited from different hospitals in different countries, including 44 healthy control samples from presurgical controls or healthy volunteers. For retrospective association with transcriptomic data, metadata with clinical and demographic parameters were collected at triage and within 72 h following ER and ICU admission.

### 2.4 Statistical analysis and machine learning methods

Preparation, processing, and analysis of the transcriptomic data were performed using the R software (version 3.6.3). Preparation, construction, evaluation, and visualization of machine learning data were performed using Python software (version 3.7).

**FIGURE 1:**
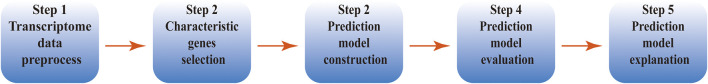
Workflow of risk prediction for the transcriptomic data based on bioinformatics and machine learning methods.

The complete flow chart for statistical analysis is shown in Figure 1. Continuous numerical variables that follow a normal distribution are described as mean ± standard deviation. When the criteria for the normal distribution were not satisfied, the data was represented by the median along with the lower and upper quartiles. For categorical variables, the data were expressed as the sum (percentage).

The R software was used for bioinformatics analysis including downloading, annotating, and organizing high-throughput data. The org. Hs.eg.db package was used for gene annotation.

Machine learning algorithms were implemented in the Python software. The data were organized in the format required for implementing the machine learning algorithm. Samples were classified into healthy or sepsis groups, defined based on the outcome indicators for the classification prediction model. The KNN algorithm (Bania and Halder, 2020) was used to fill in the missing data. The preprocessed data set was divided into training and test sets in a ratio of 2:1 (274 for the training set and 118 for the test set) (Fabian et al., 2011; Yang et al., 2022).

Features that do not follow a standard normal distribution may cause the estimators in machine learning to deliver faulty outcomes. In our study, we used the StandardScaler utility class from the preprocessing module to perform data standardization. Recursive feature elimination (RFE) can be used for feature selection to improve estimators’ accuracy scores or to boost their performance on very high-dimensional datasets. In our study, logistic regression (penalty = “L2”, c = 0.01) was used as an external estimator, which assigns weights to features. The goal of RFE was to select features by recursively considering increasingly smaller sets of features. As the desired number of features was set to 10, the procedure was recursively repeated on the pruned set until the 10 best features were selected.

The sklearn (scikit-learn) package (Fabian et al., 2011), a simple and efficient tool for predictive data analysis, was used for the prediction model, compared with traditional methods of linear regression and Lasso regression. The 10-fold cross-validation was used to prevent overfitting. The calibration curve of the models and decision curve analysis (DCA) were used to classify the calibration models.

Shapley Additive explanation (SHAP) package (Lundberg and Lee, 2017), a method for uniform measure of the feature importance in machine learning models, was used for the visualization and explanation of the prediction model. Explanations based on the Shapley value have a solid theoretical foundation; it is the only attribution method that satisfies the requirements of local accuracy, missingness, and consistency (Lundberg et al., 2020; Yang et al., 2022). However, the Shapley value provides only a local explanation (Yang et al., 2022). The SHAP package developed by Lundberg et al. based on the Shapley value inherits all of the advantages of the Shapley value (Lundberg and Lee, 2017; Yang et al., 2022). Making the global analysis consistent with the local explanation, SHAP has been used to perform interpretability analysis by different researchers in their respective fields (Lundberg and Lee, 2017; Lundberg et al., 2020). The SHAP force plot was used to illustrate the model at the individual level.

## 3 Results

### 3.1 Preprocessing of the transcriptomic data

The counts for the expression matrix were downloaded locally from GEO (GSE185263) (Baghela et al., 2022). The downloaded expression matrix was filtered using the ensemble_id and SYMBOL fields and the org. Hs.eg.db package. A total of 29,663 successfully annotated data points using the SYMBOL field were obtained. Taking the SYMBOL variable as a reference, duplicates (retaining the first duplicate), and missing values (>50%) were removed, and a total of 26,530 successfully annotated transcriptomic expression matrices were finally obtained.

The gene expression matrix was inverted using the annotated variables with SYMBOL as the column name and the sample name as the row name. Variables with missing values greater than a proportion of 30% in the data annotated as SYMBOL was removed. The resulting dataset was used for implementing the machine learning algorithms.

### 3.2 Selection of characteristic genes

The data was organized in the format required for implementing the machine learning algorithm. The KNN algorithm (Bania and Halder, 2020) was used for filling in missing data. Recursive feature elimination (RFE) was used for feature selection to improve estimators’ accuracy scores or to boost their performance on very high-dimensional datasets. Logistic regression (penalty = “L2”, c = 0.01, n = 10) was used as an external estimator which assigns weights to features.

### 3.3 Construction of prediction models

Traditional methods of linear regression and Lasso regression were used first. The scores of the training and test sets were 0.66 and 0.53 in linear regression, respectively. Similarly, the scores of the training and test sets were 0.66 an 0.53 in Lasso regression, respectively. (Table 1).

**TABLE 1:** Methods used for models and the classification report of the test set.

			Test set					
	Scores		Precision		Recall		f1-scores	
	Train set	Test set	Normal	Sepsis	Normal	Sepsis	Normal	Sepsis
Linear	0.66	0.53						
LASSO Linear	0.66	0.53						
Logistic	0.99	0.94	0.69	0.97	0.75	0.96	0.72	0.97
Decision Tree	1.0	0.94	0.69	0.97	0.75	0.96	0.72	0.97
Random Forest	1.0	0.96	0.69	0.97	0.75	0.96	0.72	0.97
ANN-MLP	0.99	0.96	0.90	0.97	0.75	0.99	0.82	0.98
CatBoost	1.0	0.97	0.90	0.97	0.75	0.99	0.82	0.98

**FIGURE 2:**
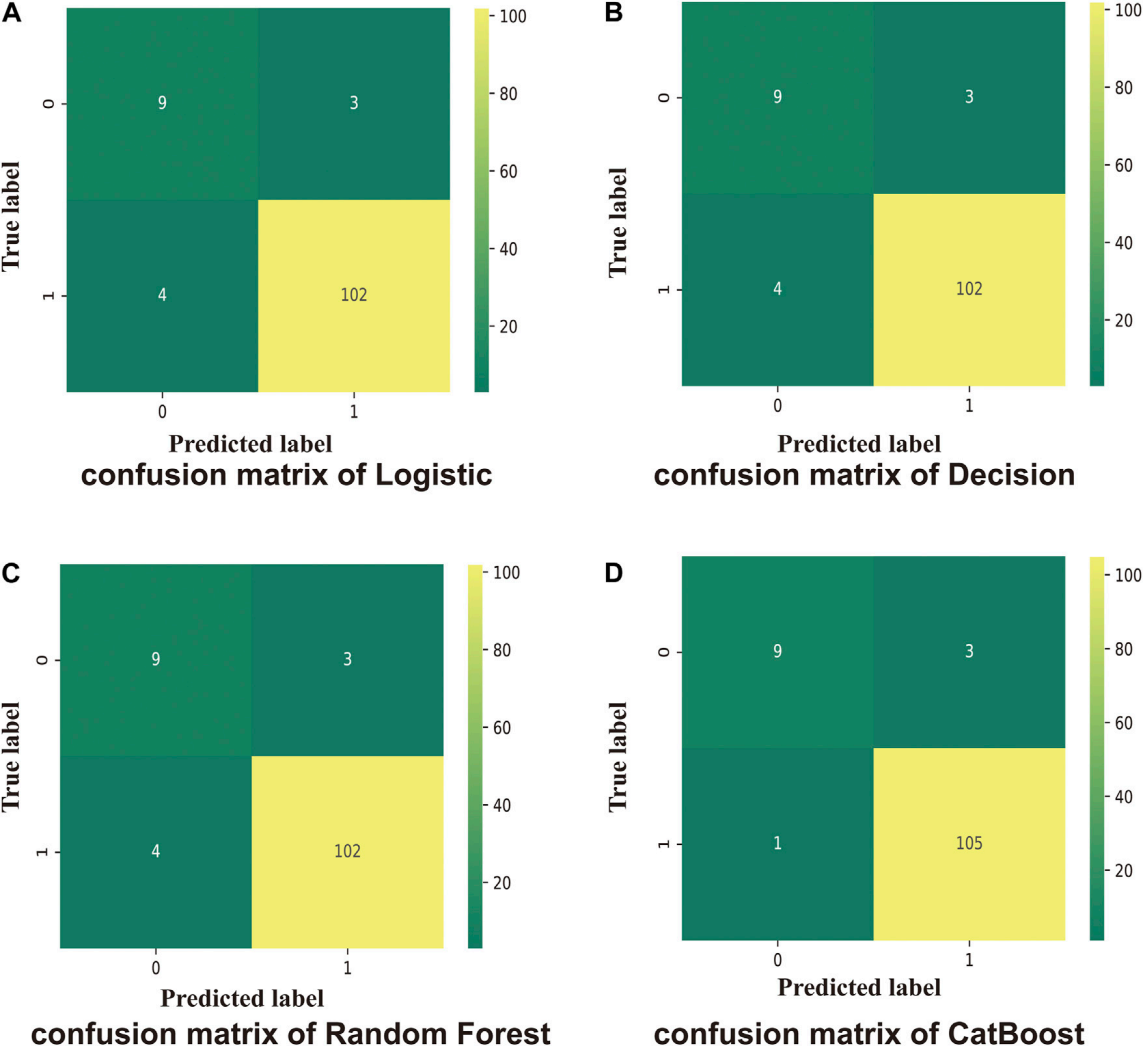
Confusion matrix. One indicates sepsis; 0 indicates healthy. Numbers are the total number of patients. **(A)** Test scores for the logistic method (0.94); 0.72 (f1-score of healthy patients), and 0.97 (f1-score of patients with sepsis). **(B)** Test score using the Decision Tree method (0.95); 0.72 (f1-score of healthy patients), and 0.97 (f1-score of sepsis patients). **(C)** Test score using the Random Forest method (0.96); 0.72 (f1-score of healthy patients), and 0.97 (f1-score of sepsis patients). **(D)** Test score using the CatBoost method (0.97); f1-score of healthy: 0.82, f1-score of sepsis: 0.98.

The logistic model was constructed using the LogisticRegression classifier in the linear_model module of the scikit-learn machine learning library (sklearn). The score of the training and test sets were 0.98 and 0.94, respectively. The results of the model trained on the training and test datasets are shown in Figure 2A for the confusion matrix. The f1-score for the healthy and sepsis groups was 0.72 and 0.97, respectively.

The Decision Tree model used was constructed using the DecisionTreeClassifier model from the sklearn. tree module. The scores of the training and test sets were 1.0 and 0.95, respectively. The results of the model for the test set are shown in Figure 2B for the confusion matrix. The f1-score for the healthy and sepsis groups was 0.72 and 0.97, respectively.

The Random Forest model was constructed using the RandomForestClassifier method from the sklearn. ensemble module. The scores of the training and test datasets were 1.0 and 0.96, respectively. The results of the model entrained on the training set for the test set are shown in Figure 2C for the confusion matrix. The f1-score for the healthy and sepsis groups was 0.72 and 0.97, respectively.

Next, the CatBoost model was constructed using the CatBoostClassifier model from the CatBoost algorithm. The scores of the training and test datasets were 1.0 and 0.97, respectively. The results of the model entrained on the training set for the test set are shown in Figure 2A for the confusion matrix. The f1-score of the healthy and sepsis groups is 0.82 and 0.98, respectively.

**FIGURE 3:**
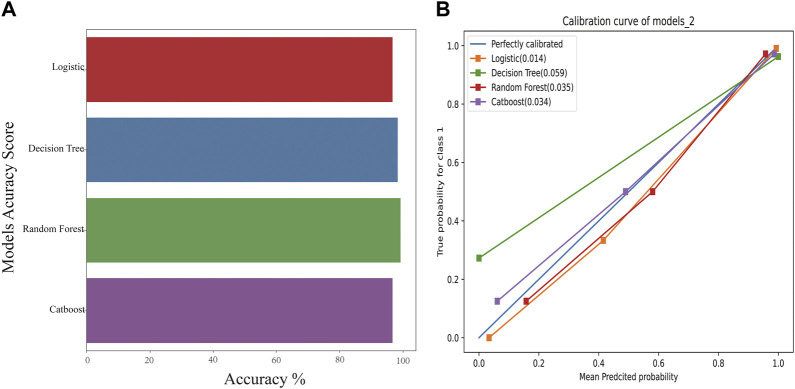
The accuracy scores of models and their calibration curves. **(A)**: Accuracy scores of models constructed using machine learning. **(B)**: The calibration curve of models is shown in Figure 3B. The diagonal line is the reference, i.e., the case where predicted value = actual value. If predicted value = observed value, the calibration curve coincides exactly with the reference. If predicted value > observed value, wherein the risk is overestimated, the calibration curve is below the reference. If the predicted value is less than the observed value, wherein the risk is underestimated, the calibration curve is above the reference.

Compared with the traditional methods, models built by machine learning had better scores. Similarly, on comparing the different models, the CatBoost model showed the best results for the test set and the f1-score (Figure 3A).

### 3.4 Evaluation of prediction models

#### 3.4.1 The calibration curve of models

The calibration curves for models are shown in Figure 3B. The diagonal line is the reference, i.e., the case where predicted value = actual value. If predicted value = observed value, the calibration curve coincides exactly with the reference. If predicted value > observed value, whereby risk is overestimated, the calibration curve is below the reference. If the predicted value is less than the observed value, whereby the risk is underestimated, the calibration curve is above the reference. As shown in Figure 3B, the predicted values from the four models exhibited good performance, with random forest and CatBoost showing the best performances (0.035 and 0.034).

#### 3.4.2 Ten-fold cross-validation to evaluate the prediction models

**FIGURE 4:**
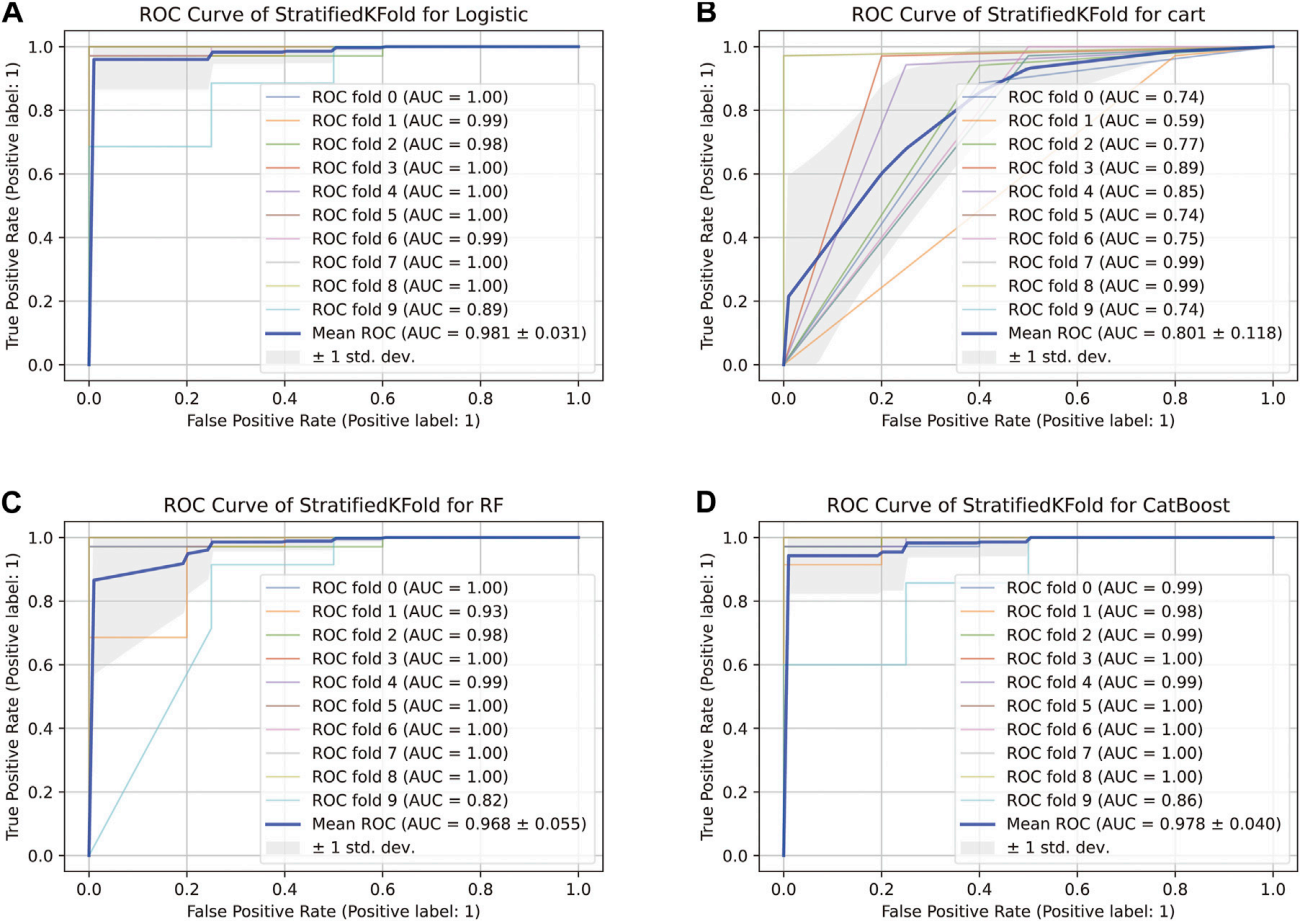
ROC curve of the stratified K-Fold analysis. **(A)** Comparison of ROC curves of the logistic model based on the tenfold cross-validation results. **(B)** Comparison of ROC curves of the Decision Tree model based on the tenfold cross-validation results. **(C)** Comparison of ROC curves of the Random Forest model based on the tenfold cross-validation results. **(D)** Comparison of ROC curves of the CatBoost model based on the tenfold cross-validation results.

The comparison of receiver operating characteristic (ROC) curves of the logistic, Decision Tree, Random Forest, and CatBoost models based on the evaluation of ten-fold cross-validation results is shown in Figures 4A–D. The area under the curve (AUC) was between 0.89 and 1.00, 0.74 and 0.99, 0.82 and 1.00, and 0.86 and 1.00, respectively (mean AUC of ROC = 0.981 ± 0.031, 0.801 ± 0.118, 0.968 ± 0.055, and 0.978 ± 0.040, respectively).

For the genomic prediction models, the k-fold cross-validation method was applied and it is a statistically powerful method to assess differences in model accuracies (Schrauf et al., 2021). The results in Figure 3 show that the CatBoost model outperformed the other models.

#### 3.4.3 DCA to evaluate the benefit of the prediction models

DCA is a method to determine whether the use of a prediction model to inform clinical decision-making would be beneficial (Vickers and Elkin, 2006; Vickers et al., 2008). In DCA, the two dotted lines, reflecting the strategies of “use the strategy on all patients” (i.e., treat all) and “use the strategy on none of the patients” (i.e., treat none) cross at the midpoint of the preference range (Vickers and Elkin, 2006). Interpretation of the decision curve consists of comparing the net benefit of the test, model, or marker between the strategies of “treat all” and “treat none” (parallel to the *x*-axis at a net benefit of zero). The strategy with the highest net benefit at a particular threshold probability is optimal, irrespective of the size of the difference (Vickers et al., 2008).

**FIGURE 5:**
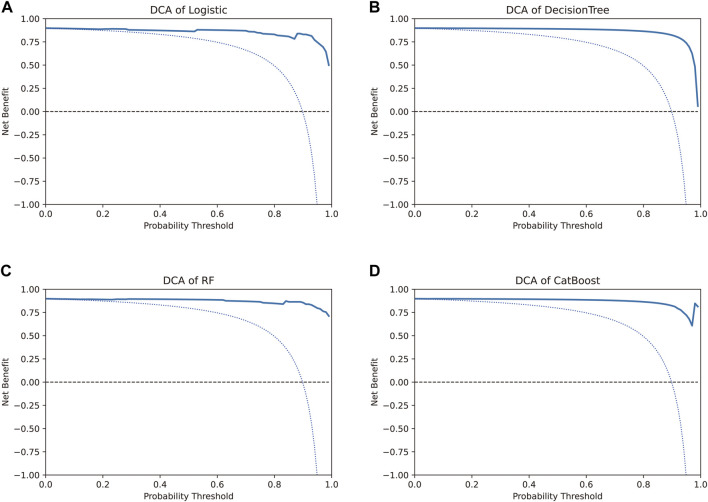
Decision curve analysis to evaluate the benefit of prediction models. The two dotted lines reflecting the strategies of “assume all patients have strategies” (i.e., treat all) and “assume no patients have strategies” (i.e., treat none) cross at the midpoint of the preference range. In the CatBoost model, a high net benefit was observed across a wide range of threshold probabilities, whereas the other three models showed a significant decrease in net benefit when the threshold probabilities were greater than 90%.

The net benefit, evaluated using the decision curve for the four models, was higher than that for either the “treat all” strategy or the “treat none” strategy, for all likely threshold probabilities (Figure 5). The three models other than the CatBoost model showed a significant decrease in net benefit when the threshold probabilities were greater than 90% (Figures 5A–C). In the CatBoost model, a high net benefit was observed for a wide range of threshold probabilities (Figure 5D). Thus, the DCA results indicated that the constructed models, especially the CatBoost model, could be used to aid clinical decision-making to improve outcomes for patients.

### 3.5 Visualization and explanation of the prediction models

The risk prediction model for detection of early sepsis using the transcriptome data-based method and the CatBoost method showed good performance in terms of model validity and clinical net benefit. However, the black-box properties of machine learning models result in a lack of model transparency, and existing explanations for the models are flawed in terms of the interpretation of the methods (Yang et al., 2022).

**FIGURE 6:**
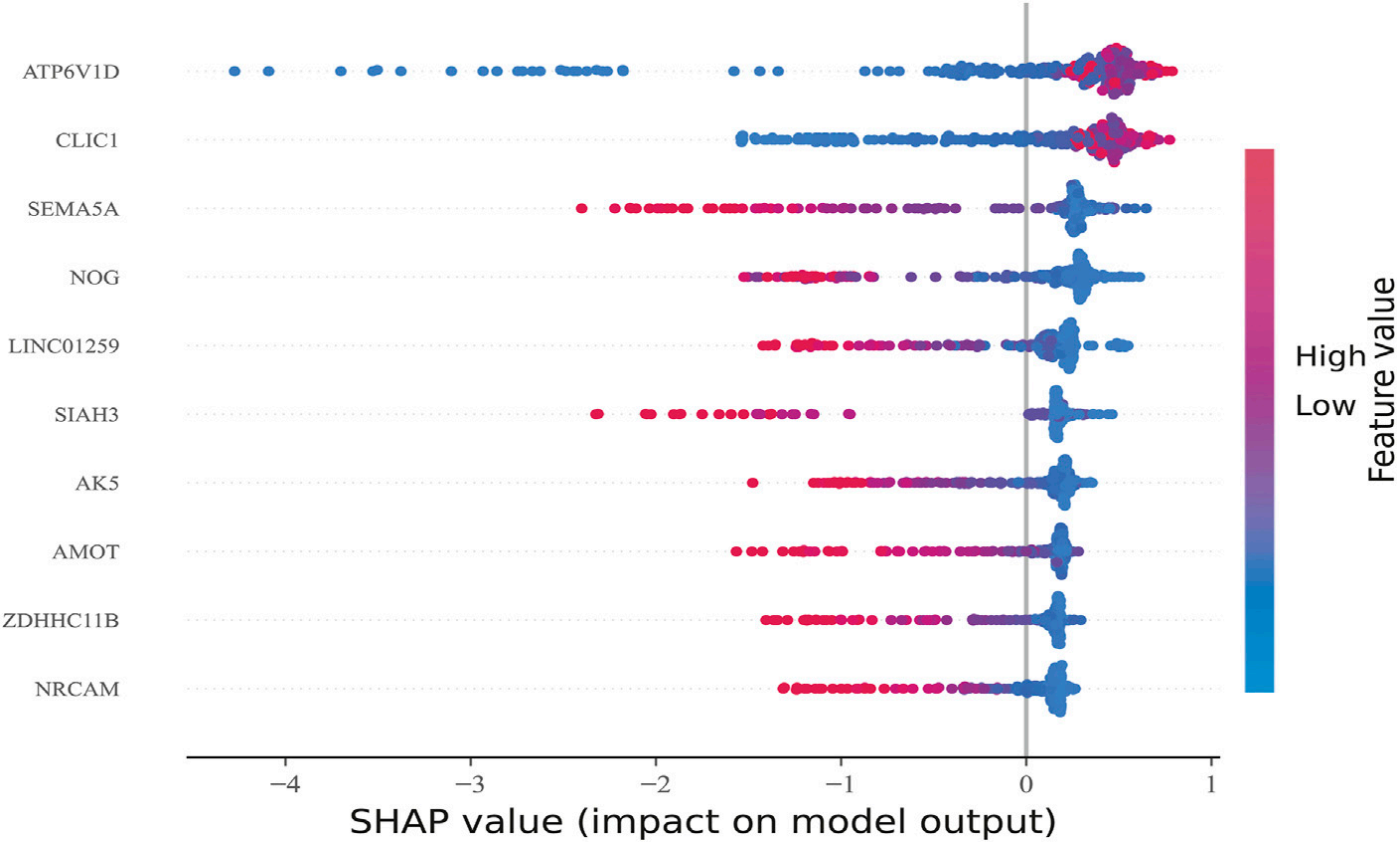
Feature Sorting: Distinguishing feature values. The vertical axis shows ranked features according to the sum of the SHAP values of all samples, and the horizontal axis indicates the SHAP value (the distribution of the influence of the features on the model output). Each point represents a sample, the sample size was stacked vertically, and the color represents the feature value (red and blue correspond to high and low values, respectively). Taking the first row as an example, we observed that high *ATP6V1D* (red) had a positive impact on prediction, and low *ATP6V1D* (blue) had a negative impact on prediction.

After sorting features, SHAP was implemented to distinguish the feature values for the selected genes. The SHAP summary plot was used to explain the model built using CatBoost. The results showed that high gene expression of *ATP6V1D* (red) had a positive impact on predictive power (Figure 6), whereas low gene expression of *ATP6V1D* (blue) had a negative impact. The results were similar to those for *CLIC1*. Other genes with high gene expression had a negative impact (red) on prediction, whereas low gene expression (blue) had a positive impact on prediction.

**FIGURE 7:**
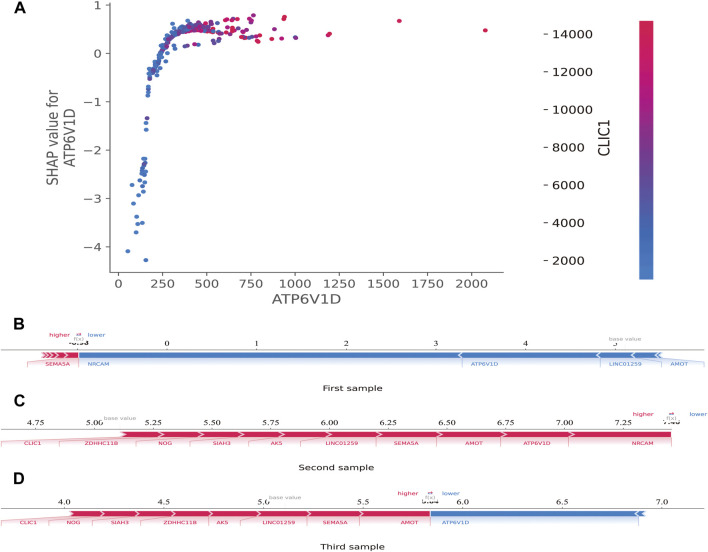
SHAP dependence and force plots. **(A)**: SHAP dependence plot. Each point represents a sample; the horizontal axis is the eigenvalue of the feature, *ATP6V1D*; the vertical axis indicates the SHAP value of the feature, *ATP6V1D*, and the color indicates the eigenvalue of the feature, *CLIC1*. For samples with higher *CLIC1* eigenvalues (red), the influence of the feature, *ATP6V1D,* was greater (the vertical axis SHAP value was higher). **(B)**: SHAP force plot. The horizontal axis is the SHAP value. Blue indicates that the feature has a negative impact on the prediction (arrow to the left, SHAP value decreases); red indicates that the feature has a positive impact on the prediction (arrow to the right, SHAP value increases). On the number axis, the base value is marked above the horizontal axis, which is the average f(x) value of all samples, and f(x) is marked above the horizontal axis, which is the average SHAP value after the samples are aggregated, that is, the model predicts the mean value. Below the horizontal axis are the key genes that influence the outcomes of this model.

Additionally, the SHAP dependence plot was used to visualize the interaction between features. In Figure 7A, each point represents a sample, with the horizontal axis indicating the eigenvalue of the feature *ATP6V1D*, the vertical axis indicating the SHAP value of the feature *ATP6V1D*, and the color indicating the eigenvalue of the feature *CLIC1*. Samples with higher *CLIC1* eigenvalues (red) showed a greater influence of the feature, *ATP6V1D*, i.e., the vertical axis SHAP value was high.

The SHAP force plot was used to illustrate the model at the individual level. The horizontal axis is the SHAP value. Blue indicates that the feature negatively impacts the prediction (arrow to the left, SHAP value decreases); red indicates that the feature has a positive impact on the prediction (arrow to the right, SHAP value increases). As shown on the number axis, the base value is marked above the horizontal axis as the average f(10) value for all samples, and f(10) is marked above the horizontal axis as the average SHAP value after the sample was aggregated, that is, the model predicts the mean value. As shown in Figures 7B–D, compared to the first sample (SHAP <0) (Figure 7B), the second (SHAP>7.25) (Figure 7C) and third (SHAP>5.5) (Figure 7D) samples belonged to high-risk groups, with a greater risk of developing sepsis. Below the horizontal axis are the key genes that influence the outcomes of this model. Different individuals may have the same or slightly different key genes that affect outcomes.

## 4 Discussion

Evans et al. and Purcell et al. have proposed methods for aggregating information related to single nucleotide polymorphisms associated with a trait. These models generate polygenic risk scores from thousands of genes to predict an individual’s genetic risk of developing disease (Purcell et al., 2007; Evans et al., 2009). By providing a predictor with discrimination properties, the polygenic score can be used to predict individual trait values or risk of disease, which is an improvement on the use of traditional established markers alone (Wray et al., 2007). The use of machine learning can improve the performance of polygenic scores by weighting the contribution of individual variants (Paré et al., 2017). Machine learning has been utilized to infer phenotypes from genomic data using changes in RNA expression levels, protein expression, single nucleotide polymorphisms, and other data types (Cheng et al., 2021). The accurate prediction of complex phenotypic traits from genomic data is both promising and challenging.

In our study, we obtained RNA expression data for sepsis at the first clinical presentation and used machine learning methods to infer disease risk from the transcriptomic data. Cheng et al. demonstrated that the integration of transcriptomic analysis and machine learning methods enhances the predictive modeling of genes affecting phenotypes (Cheng et al., 2021). We evaluated the transcriptomic data to predict the risk of sepsis in patients early by combining the data with early clinical symptoms and machine learning.

Gene expression data have been increasingly analyzed using machine learning. Due to the “large *p*, small n” paradigm of limited biological samples and the prevalence of high-dimensional data, the selection of genes is a challenging problem (Diao and Vidyashankar, 2013). Heuristic rules are utilized by most of the existing feature selection algorithms. RFE is a heuristic feature screening framework, which works by recursively removing features with the lowest weights, and has been widely used to select biological biomarkers (Guo et al., 2014; Peng et al., 2021). In our study, given an external estimator (LogisticRegression, penalty = "L2″, C = 0.01), the procedure was recursively repeated on the pruned set of genes until the desired ten features intended for selection were reached, and these included *ATP6V1D*, *CLIC1*, *SEMA5A*, *NOG*, *LINC01259*, *SIAH3*, *AK5*, *AMOT*, *ZDHHC11B*, and *NRCAM*. It is possible to set the number of genes (num = n) to be screened according to specific requirements to enable RFE to identify the optimal number of features using a cross-validation cycle.

Katsaouni et al. have described commonly used metrics in machine learning (they include metrics used in at least two studies), including logistic regression, random forests, support vector machines, deep neural networks, and convolutional neural networks (Katsaouni et al., 2021). Due to differences in the diseases investigated, it is difficult to compare the effectiveness of the machine learning methods. There is no clear favorite method that can be used as the optimal solution in all studies. CatBoost is a novel machine learning method with two innovations: Ordered Target Statistics and Ordered Boosting (Hancock and Khoshgoftaar, 2020). Studies have shown that CatBoost had the best results among all classifiers for all metrics except for specificity for data from diverse datasets including that for medicine, biochemistry, meteorology, and others (Hancock and Khoshgoftaar, 2020; Zhang et al., 2021). In our study, the CatBoost model showed the best results for the test set and the f1-score.

Schrauf et al. (Schrauf et al., 2021) showed that k-fold cross-validation is a generally applicable and powerful method to assess differences in model accuracies. The method can be used to accurately simulate the usage of the genomic model, with high statistical power. In our study, the AUC of the CatBoost model was between 0.86 and 1.00 (mean AUC of ROC = 0.978 ± 0.040), and the model performed better than the others evaluated.

The traditional biostatistical approach to evaluating models focuses on the results for sensitivity, specificity, and AUC. These methods are mathematically simple to implement but have low clinical relevance. Studies have shown that discrimination by AUC is insufficient (Van Calster et al., 2019; Vickers and Holland, 2021); a model tells a patient how well their personal risk can be distinguished from another patient’s with high discrimination, but not whether the actual risk given by a model is accurate. DCA was developed to overcome the limitations of traditional biostatistical methods and can help evaluate whether using a model to aid clinical decision-making would improve outcomes for patients and whether genomic profiling would be clinically relevant (Ho-Le et al., 2021). In our study, DCA helped analyze whether the various models could improve predictive outcomes for the patients in our study, especially for the CatBoost model.

Artificial intelligence methods based on machine learning yield black-box models. Compared with traditional models, these black-box models have shown overwhelming advantages in obtaining accurate predictions (Yang et al., 2022). In principle, it is important to use “explainable models”; however, the machine learning models currently in use are claimed to be explainable but are not explainable or quasi-explainable (Yang et al., 2022). These models can provide flawed explanations in some studies that can lead to flawed correlation conclusions (Haufe et al., 2014). Lundberg et al. developed the SHAP model, which uses the SHAP value as a uniform measure of the importance of features used in the machine learning models (Lundberg et al., 2020). By attributing output values to the Shapley value of each feature, researchers have performed interpretability analysis of machine learning models (Wang et al., 2021; Wojtuch et al., 2021; Scavuzzo et al., 2022). In this study, high gene expression of *TP6V1D* had a positive impact on prediction, whereas low gene expression of *ATP6V1D* negatively impacted prediction, similar to *CLIC1*. Other genes with high expression had a negative impact on prediction, whereas low gene expression had a positive impact on prediction. The results of the interpretability analysis demonstrate the excellent applicability and generalizability of the findings obtained using the CatBoost model.

The SHAP force plot illustrated the model at the individual level. Through SHAP analysis of individual samples, we screened the high-risk samples and identified the high-risk patients. If these analyses are extended to identify transcriptomic biomarkers associated with disease severity within the first few hours of ER or ICU admission, timely and aggressive interventions to prevent further development of severe sepsis may be prevented.

Our study had several innovations. It was the first to implement the CatBoost machine learning approach applied to a polygenic risk scoring model. In comparisons using the same dataset, we showed that CatBoost was more efficient than other machine learning methods and showed better accuracy and validity. This study was also the first where SHAP was applied to a polygenic risk scoring model constructed based on machine learning to explain the model. This study was also the first wherein SHAP was applied in a machine learning model for patients with sepsis. The visualization of SHAP values helped explain the constructed risk prediction model. This study was the first attempt at constructing a polygenic risk assessment model using machine learning combined with SHAP scores in critically ill patients. Our analysis is by far the most integrated polygenic risk assessment process for transcriptomic data and can be extended to the analysis of other omics datasets.

This study has a few limitations. First, the data was obtained from a public database, and the number of patients in the control group was relatively small. Second, our results have been evaluated using machine learning methods alone; further validation using molecular and clinical analyses is required. Our study can be used as a model for exploring genes related to disease occurrence or prognosis risk in transcriptomics or genomics, by expanding the RFE value in the second step if necessary to expand the screening scope of candidate genes. In later research, we will adopt a prospective multi-center randomized control design, and further expand the sample size. Combining this strategy with cohort studies, we will explore the relationship between the early expression levels of the above-mentioned candidate genes and the clinical prognosis.

## 5 Conclusion

We describe herein an application based on bioinformatics, machine learning, and SHAP for risk prediction among patients with risk of sepsis at the first clinical presentation using transcriptomic data analysis. The process included preprocessing of sequencing data, screening of several genes, construction of the machine learning prediction model, its validation, visualization, and interpretation. The combination of CatBoost and SHAP may serve as an optimal machine learning model for transcriptomics and predicting disease risk. Extending these analyses to identify transcriptomic biomarkers associated with disease severity within the first few hours may enable timely and aggressive interventions to prevent progression to severe sepsis. The workflow may provide a new approach and direction for elucidating mechanisms associated with genes and phenotypes correlated with diseases.

## Data Availability

The original contributions presented in the study are included in the article/supplementary material further inquiries can be directed to the corresponding authors.

## Abbreviations

SHAP, Shapley Additive explanation; GEO, Gene Expression Omnibus; KNN, K-Nearest Neighbor; RFE, Recursive feature elimination; DCA, Decision curve analysis; ROC, receiver operating characteristic; AUC, area under the curve
